# Toxicity and Repellency Efficacy of Benzyl Alcohol and Benzyl Benzoate as Eco-Friendly Choices to Control the Red Flour Beetle *Tribolium castaneum* (Herbst. 1797)

**DOI:** 10.3390/molecules28237731

**Published:** 2023-11-23

**Authors:** Shawky M. Aboelhadid, Samar M. Ibrahium, Heba Abdel-Tawab, Ahmed O. Hassan, Saleh Al-Quraishy, Fatma El-zahraa R. Saleh, Abdel-Azeem S. Abdel-Baki

**Affiliations:** 1 Parasitology Department, Faculty of Veterinary Medicine, Beni-Suef University, Beni-Suef 62511, Egypt; 2 Parasitology Department, Animal Health Research Institute, Fayum Branch, Fayum 16101, Egypt; drsamarmahmoud333@yahoo.com; 3 Zoology Department, Faculty of Science, Beni-Suef University, Beni-Suef 62511, Egyptaabdelbaki@science.bsu.edu.eg (A.-A.S.A.-B.); 4 Department of Medicine, Washington University School of Medicine, St. Louis, MO 63110, USA; 5 Zoology Department, College of Science, King Saud University, Riyadh 11451, Saudi Arabia; 6 Water Pollution Research Department, National Research Centre, Giza 12622, Egypt

**Keywords:** bio-insecticide, benzyl alcohol, benzyl benzoate, *Tribolium castaneum*, oxidative stress, acetylcholinesterase

## Abstract

*Tribolium castaneum* is a damaging pest of stored grains, causing significant losses and secreting lethal quinones, which render the grains unfit for human consumption. Chemical insecticides are the most commonly used approach for control; however, they create insecticide resistance and affect the health of humans, animals, and the environment. As a result, it is critical to find an environmentally friendly pest-management strategy. In this study, two naturally occurring chemicals, benzyl alcohol (BA) and benzoyl benzoate (BB), were investigated for insecticidal activity against *T. castaneum* using different assays (impregnated-paper, contact toxicity, fumigant, and repellency assays). The results showed that BA had a significant insecticidal effect, with the LC_50_ achieved at a lower concentration in the direct-contact toxicity test (1.77%) than in the impregnated-paper assay (2.63%). BB showed significant effects in the direct-contact toxicity test, with an LC_50_ of 3.114%, and a lower toxicity in the impregnated-paper assay, with an LC_50_ of 11.75%. Furthermore, BA exhibited significant fumigant toxicity against *T. castaneum*, with an LC_50_ of 6.72 µL/L, whereas BB exhibited modest fumigant toxicity, with an LC_50_ of 464 µL/L. Additionally, at different concentrations (0.18, 0.09, 0.045, and 0.0225 µL/cm^2^), BA and BB both showed a notable and potent repelling effect. BA and BB significantly inhibited acetylcholinesterase, reduced glutathione (GSH), and increased malondialdehyde (MDA) in treated *T. castaneum*. This is the first report of BA insecticidal activity against the red flour beetle. Also, the outcomes of various assays demonstrated that the application of BA induces a potent bio-insecticidal effect. BA may be a promising eco-friendly alternative to control *T. castaneum* due to its safety and authorization by the EFSA (European Food Safety Authority).

## 1. Introduction

*Tribolium castaneum* (Coleoptera, Tenebrionidae) is known to destroy a variety of stored grains [1]. According to Ajayi and Rahman [2], a *T. castaneum* infection is responsible for 40% of the weight loss of wheat flour each year. This beetle also secretes toxic quinones that are carcinogenic and pose a major risk to human health [3]. These characteristics contribute to the ongoing challenge in controlling *T. castaneum* infestations and the implications for both grain market economics and nutritional quality [4]. Furthermore, *T. castaneum*’s resistance to pesticides is reinforced by potentials such as sexual selection by females for the capacity of the progeny [5,6], quick adaptation to a new environment [7], and rapid dispersal to establish new feeding patches. As a result, the quality of grains kept in storage facilities is a major concern for all countries. Fumigation with methyl bromide and phosphine is the only control method for a variety of stored grain pests [8]. However, the use of methyl bromide has been outlawed globally due to its direct connection to ozone layer weakening [9,10]. Moreover, phosphine was found to be the least efficient in getting rid of a variety of stored grain pests, including *T. castaenuem*, due to the beetle’s quick development of resistance [11]. Therefore, developing natural, eco-friendly approaches to control *T. castaenuem* is crucial for both economic and health concerns [12,13]. Benzyl alcohol (BA) is a naturally occurring monoaromatic alcohol with a high polarity, low volatility, and negligible toxicity [14]. BA is a safe and efficient solvent that is used with polymers to produce inks, glues, paints, and hardening products such as epoxy resins [15,16,17]. In addition, BA is used in the manufacture of cosmetics [18] and food products [19]. BA has a bacteriostatic activity at low concentrations [20] and is utilized as a topical agent and preservative [21,22]. Furthermore, BA is naturally occurring in numerous plants, including tea leaves and the essential oils of ylang-ylang, jasmine, and hyacinth [23]. A recent study found that BA has acaricidal activity against different stages of *Rhipicephalus annulatus* (Say) and *R. sanguineus* ((Latreille) (Acari: Ixodidae)) [24]. It has been used to treat pediculosis and scabies [25]. It was also found that benzyl alcohol is safe to use in hair dyes at concentrations of up to 10% [26]. Benzyl benzoate (BB) is an organic compound that is an active ingredient in essential oils such as Peruvian groundcherry (*Physalis peruviana*) and Toulu balsam (*Myroxylon balsamum*); it can also be produced synthetically and sold as a liquid, emulsion, or lotion. [27]. BB is used as a medication, as an insect repellent, and to eradicate scabies and lice [28]. BB is effective at controlling a variety of mites, including *Tyrophagus putrescentiae* (Sarcoptiformes, Acaridae) [29], *Dermatophagoides pteronyssinus* (Sarcoptriformes, Pyroglyhidae) [30,31], and *Dermutophagoides furinue* (Astigmata, Pyroglyphidae) [32]. A topical application of BB + crotamiton was found to be effective for treating rosacea and demodicosis [33,34]. Forton et al. [35] reported that BB has acaricidal effects on *Demodex folliculorum* (Trombidiformes, Demodecidae). BB is also used as a pediculicide and scabicide in dogs, but its principal purpose in humans is as a tick, chigger, and mosquito repellent [36]. Therefore, evaluating the potential role of benzyl alcohol and benzyl benzoate in the control of the red flour beetle, *T. castaneum*, was the aim of the current investigation.

## 2. Results

### 2.1. Contact Toxicity Bioassays of Benzyl Alcohol and Benzyl Benzoate against Adult Red Flour Beetles

BA exhibited a significant toxicity in both methods of application (Table 1). It was found that the LC_50_ value of BA in the direct-contact toxicity assay was lower than that in the impregnated-filter-paper assay (1.90% vs. 2.63%) (Table 1). In the filter paper assay, BB exhibited a mild toxic effect on *T. castaneum* (Table 1); however, in the direct-contact toxicity assay, BB showed a significant toxicity, with an LC_50_ of 4.17%. Furthermore, BA was more toxic than BB, notably in the filter paper assay, with the LC_50_ attained at a concentration of 2.63% for BA and 11.7% for BB (Table 1).

### 2.2. Fumigant Toxicity of Benzyl Alcohol and Benzyl Benzoate against Adult Red Flour Beetles

BA exhibited a high fumigant toxicity against *T. castaneum*, with LC_50_ and LC_90_ values of 6.72 µL/L and 23.6 µL/L, respectively (Table 2), while BB induced a minor fumigant impact, with LC_50_ and LC_90_ values of 464 and 822 µL/L, respectively.

### 2.3. Persistence of Toxicity of Benzyl Alcohol and Benzyl Benzoate at LC_90_ against Tribolium castaneum

The persistence of toxicity was significantly affected by the number of days (F9, 108 = 352.5, *p* < 0.001), and this effect was qualified by a significant day*group interaction (F18, 108 = 114.846, *p* < 0.001) (Table 3). BA exhibited toxicity against *T. castaneum* until day 5, following which, the toxicity decreased and reached its lowest efficacy after day 9 (Table 4). The BB toxicity, on the other hand, rapidly decreased and reached its lowest efficacy by day 7 (Table 4). However, when compared to BA and the control, BB showed a significant mean for the persistence of its toxicity (*p* < 0.05).

### 2.4. Repellent Effect of Benzyl Alcohol and Benzyl Benzoate against Adult Red Flour Beetles

**Figure 1:**
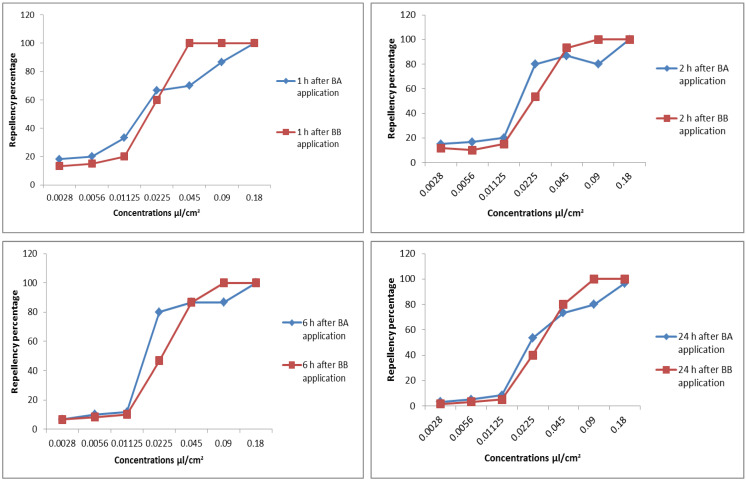
Repellency percentage of benzyl alcohol and benzyl benzoate against *Tribolium castaneum* at different periods (1, 2, 6, and 24 h post-application).

The concentration of benzyl alcohol significantly affected its repellency against red flour adults (F6, 56 = 71.701, *p* < 0.001), but not the time (F3, 56 = 2.384, *p* = 0.079), and the interactions had no effect (Figure 1). At a high concentration (0.0225 µL/cm^2^), about 50% of the insects were repelled. The efficacy of the repellent was significantly diminished at a low concentration of 0.0122 µL/cm^2^ (Figure 1). The concentration (F6, 56 = 401.523, *p* < 0.001) and time (F3, 56 = 8.547, *p* < 0.001) had a significant effect on the repellent effect of BB; however, the effects of the interactions were not significant. At a 0.0122 µL/cm^2^ concentration, BB exhibited very little repellent action. In contrast, over 80% of the insects were repelled by BB at a concentration of 0.045 µL/cm^2^, and over a period of 24 h, 100% of the insects were repelled at concentrations of 0.09 µL/cm^2^ and 0.18 µL/cm^2^. Additionally, over time, BB’s ability to repel insects diminished (Figure 1).

### 2.5. Biochemical Alterations in the Treated Insect

**Figure 2:**
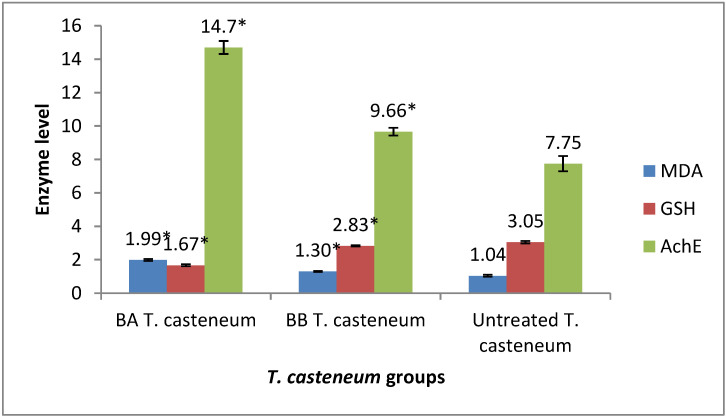
Effects of benzyl alcohol and benzyl benzoate treatments on the oxidative/antioxidative and acetylcholinesterase inhibition against *Tribolium castaneum*, treated by LC_90_ through a direct-contact bioassay. (*) significant compared to untreated group.

The lethal dose of BA and BB that killed 90% of *T. castaneum* induced significant oxidative stress by increasing the MDA levels and decreasing the GSH levels (*p* ≤ 0.05) (Figure 2). Furthermore, the increase in MDA levels in the BA-treated group was greater than in the BB-treated group (*p* ≤ 0.05). Both BA and BB altered the neurotransmission function through the inhibition of AchE activity in the treated pest (Figure 2).

## 3. Discussion

The flour beetle, *Tribolium castaneum*, is an important economic pest due to its widespread distribution and significant impact on the quantity and quality of stored grain [1,37]. Furthermore, the genus *Tribolium* has the potential to exhibit significant adverse effects on human health due to the release of several toxic quinones that have carcinogenic properties [3]. Traditional management approaches for *T. castaneum* and other stored product beetles are becoming less effective. This is largely due to insect populations developing resistance to pesticides, their residual toxicity in food grains, their impact on non-target species, and other negative environmental consequences [38]. Nowadays, biological control is more frequently used to manage pests of stored cereals because it is environmentally friendly with no adverse impacts [39,40]. Benzyl alcohol is mostly used in pharmaceuticals, as either an injectable or topically (EMA/CHMP/272866/2017). Benzyl alcohol and benzyl benzoate are reported to be used as ingredients in fragrance, pesticides, pH adjusters, preservatives, solvents, and/or viscosity-reducing agents in the cosmetic, agricultural, and pharmaceutical industries [41,42].

This study investigated the toxicity of benzyl alcohol and benzyl benzoate at different concentrations against *T. castaneum*. The results showed a significant toxicity, with contact-method LC_50_ values for BA and BB that were lower than those obtained using the impregnated-paper method (1.77 and 2.63% vs. 3.11 and 11.7%, respectively). BA was found to be a more effective fumigant than BB, with LC50 values of 6.72 µL/L and 464 µL/L, respectively. BA and BB exhibited significant repellent efficacy after 24 h of treatment at various concentrations (0.18, 0.09, 0.045, and 0.0225 µL/cm^2^). The BA results indicated a significant toxicity against *T. castaneum*, which is supported by our recently published article on BA acaricidal activity against hard ticks [24]. BA’s insecticidal efficacy has been proven through clinical studies, which revealed that a 5% benzyl alcohol lotion is a safe and effective topical therapy for head lice [22]. Furthermore, Johnson et al. [26] proposed that a 5% benzyl alcohol lotion could be used as a significant pediculicide treatment rather than existing approved pesticides and ineffective home treatments. Sangaré et al. [25] also reported that benzyl alcohol has been effectively used at concentrations ranging from 10% to 30% to treat scabies and pediculosis. Moreover, 5% benzyl alcohol lotion, the only pediculicide approved by the FDA, has been evaluated and shown to be safe for newborns as young as six months old. It also poses no risk to pregnant women [22]. In developing countries, 10% benzyl benzoate has been used to treat scabies [43]. It has been proven that benzyl benzoate possesses significant acaricidal [44] and insecticidal [45] activities. Harju et al. [29] studied the effect of BB on *Tyrophagus putrescentiae* mites and found that spraying BB resulted in over 90% mite mortality within 20–30 min. Diastuti et al. [46] also showed that BB displayed the most potent antibacterial activity against Bacillus cereus, with an MIC of 50 g/mL and an inhibition zone of 5.9 mm. Suhaili and Ho [47] discovered that, 24 h after application, benzyl benzoate induced 100% mortality in *Dermatophagoides pteronyssinus*. Monteiro et al. [48] found that essential oil extracted from *Cinnamomum verum* leaves with benzyl benzoate as the main ingredient (65.4%) showed significant acaricidal action against *Rhipicephalus microplus*.

Regarding biochemical changes, it was found that the application of BA and BB significantly increased the MDA levels and significantly decreased the GSH levels, thereby interfering with the insect’s oxidative/antioxidative activity. Furthermore, BA and BB significantly inhibited the AchE activity in the treated insects. In line with the findings of this investigation, Aboelhadid et al. [24] found oxidative stress and the suppression of AchE activity in BA-treated ticks. The results showed that BA and BB interfered with *T. castaneum*’s nervous system by inhibiting AChE activity and altering the levels of detoxifying enzymes such as GST and MDA. As a result, the treated insects were unable to detoxify the effects of the BA and BB treatments. These factors could result in the death of the insects at lethal concentrations. Deb and Kumar [49] observed comparable results in red flour beetles treated with *Artimesia anuna*. According to Rajashekar et al. [50], AchE inhibition can be the reason for insect death. Additionally, the GST activity is suggested to be higher in multiresistant strains of *T. castaneum* [51]. The MDA levels, on the other hand, increase significantly in treated insects, indicating oxidative stress in the beetle [52]. Furthermore, Meinking et al. [22] postulated that the insecticidal mode of action of BA involves asphyxiation, as in the treatment of lice. Additionally, scanning electron microscopy revealed that 5% BA clogged the honeycomb structure of the spiracles. Yano et al. [53] claimed that BA works on the bacterial surface by inactivating membrane proteins and promoting cell penetration. Furthermore, the fact that BA can inhibit AchE and GSH suggests that BA may play a role in insect intoxication via the accumulation of AchE and free radicals.

## 4. Materials and Methods

### 4.1. Chemicals

**Figure 3:**
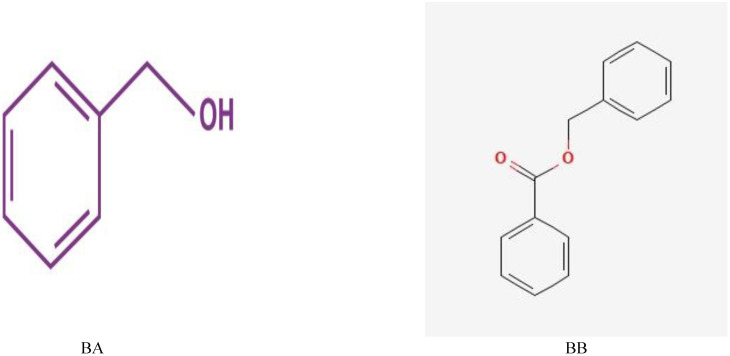
Formula of benzyl alcohol (BA) and benzyl benzoate (BB).

Benzyl alcohol (99.9% purity), benzyl benzoate (98%) (Figure 3), and acetone were purchased from the Loba Company, India. Different concentrations of BA and BB were prepared through dilution in acetone.

### 4.2. Tribolium castaneum Colony

This colony has been reared without treatment at the insectary division of our Parasitology Department laboratory at Beni-Suef University in Egypt since March 2019. The insects were reared in wheat (flour and grains in a ratio of 6:3) and yeast media. The maintained incubator had a temperature of 28 ± 2 °C and a humidity of 70 ± 5 RH. All trials were conducted at 28 ± 2 °C and 70 ± 5 RH [54].

### 4.3. Contact Toxicity Bioassays

#### 4.3.1. Impregnated-Paper Assay

The impregnated-paper assay was performed by following the method of Kljajic and Peric [55] and FAO [56]. Filter papers, Whatman No. 1 (9 cm in diameter), were submerged in various concentrations (5, 2.5, 1.25, 0.625, 0.312, 0.156, 0.078, and 0.039%) of BA and BB for 30 s. Then, the treated filter papers were placed one by one in several Petri dishes and allowed to air dry for two to three minutes. Adults of *T. castaneum* were then successively released into the Petri dish. Acetone-soaked filter paper was used as a control. Each treatment was carried out in five replicates, each with 20 adult insects. All treatments were kept in an incubator at 28 ± 2 °C with a humidity of 70 ± 5 RH. After 24 h following their release, the insects were softly stroked with a hairbrush to determine whether they were still alive. If the insect did not respond, it was considered dead.

#### 4.3.2. Contact Toxicity of BA and BB with Wheat Flour

BA and BB concentrations of 50, 25, 12.50, 6.25, 3.12, 1.56, 0.78, and 0.39 mg/g were added to wheat flour. In a 100 mL glass jar, these concentrations were combined with 10 g of wheat flour. The treated flour was shaken for 10 min in a rotary shaker to ensure that the treated material was evenly distributed throughout the flour. The treated jars were left at room temperature to ensure that the solvent was evaporated. For each jar, 20 unsexed *T. castaneum* were introduced, and the jar was then covered with muslin [57,58]. Jars containing untreated wheat flour served as a control group. Three replicas were used for each concentration. The treated jars were kept in an incubator with a temperature of 28 ± 2 °C and a relative humidity of 70 ± 5 RH. After 24 h, the number of dead insects was recorded for each treatment.

### 4.4. Fumigant Toxicity of BA and BB against T. castaneum Adults

Different concentrations of BA and BB were tested (300 µL/L, 150, 75, 35, 20, 10, 5, and 2.5 µL/L). Filter papers with a diameter of 2 cm were glued to the inside surface of 50 mL vial caps. Each paper was covered with the tested concentrations of BA and BB using a micropipette. Twenty adult insects were placed on the filter paper of each vial cap, and then the caps were covered with a net. Filter papers treated with pure acetone were used as a control group. Each concentration was repeated five times. After that, the vial caps were kept at 28 ± 2 °C, 760 ± 5% relative humidity, and a photoperiod of 16:8 h (L:D). After 24 h, the number of dead insects was counted [59].

### 4.5. Persistence of Activity of BA and BB against T. casteneum

The persistence of the insecticidal effect of BB and BA was investigated using the Ilboudo et al. [60] method. In brief, ten 250 mL glass jars containing 20 g of wheat flour were prepared before being treated with LC_90_ concentrations of BA (3.8%) and BB (7.64%). Then, twenty *T. casteneum* adults were inserted into each jar. The mortality was observed after 24 h. The insects in each jar, whether dead or alive, were then replaced with new ones, and the mortality was reported after 24 h, and so on.

### 4.6. Repellency Using Filter Paper Area Test

The repellency of BA and BB was assessed according to Cosimi et al. [61]. Different concentrations of BA and BB (0.18, 0.9, 0.045, 0.022, 0.011, 0.0056, and 0.0028 µL/cm^2^) were prepared. Filter papers with a 9 cm diameter were divided into 2 halves. Half of the filter paper was treated with a prepared concentration of BA or BB, while the other half was treated with acetone as a control. Cellotape was used to attach both pieces of filter paper to the underside of the Petri dish after being dried for two minutes. Ten adults of both sexes were released onto the center of the Petri dish. Each concentration was tested using five replicates. The results were recorded at 1, 2, 6, and 24 h intervals. The following formula was used to calculate the % repellency from the raw data: PR = 2(C − 50), where C is the percentage of insects recorded on the untreated half of the filter paper. Positive numbers indicated repellency, while negative ones indicated attraction. This assay was performed at room temperature.

### 4.7. Determination of the Biochemical Alterations in the Treated T. castenuem

Insects treated with the LC_90_ of BA and BB via a direct-contact toxicity assay (50 insects each) were homogenized in lysis buffer for 5 min using a mortar in 100 mM sodium phosphate buffer containing a protease inhibitor. Subsequently, the homogenizations underwent a 10 min centrifugation at 10,000 rpm and 4 °C. Following centrifugation, the fluids in the supernatant were aspirated using an automated pipette and kept until the levels of malondialdehyde (MDA), reduced glutathione (GSH), and acetylcholinesterase (AchE) were determined [62].

#### 4.7.1. Acetylcholinesterase (AchE) Inhibition

The AchE activity was detected using a modified version of the Ellman et al. [63] method. Anderson and Coats [64] estimated the AchE inhibition according to the following equation:

$$\mathrm{AchE}\;\mathrm{inhibition}\;(\%)=100-[\frac{\mathrm{As}}{\mathrm{Ac}}\;\times \;100],$$

where As = AChE activity for each treatment and Ac = AChE activity for the negative control.

#### 4.7.2. Reduced Glutathione (GSH) Activity Assay

The GSH activities were assessed according to Tavares et al. [65]. Trichloroacetic acid was thoroughly mixed with 0.5 mL of tick extract and left to stand at room temperature for 5 min. This mixture was centrifuged at 3000 rpm for 15 min. The supernatant was then thoroughly mixed with buffer and 100 µL of DTNB (5,5′ dithiobis (2-nitrobenzoic acid). After 5–10 min, the absorbance at 405 nm was measured.

#### 4.7.3. Malondialdehyde (MDA) (Lipid Peroxide)

The MDA levels were determined by following the method of Bar-Or et al. [66]. The interaction of MDA with thiobarbituric acid led to the creation of a red-colored compound; then, the absorbance at 532 nm was measured.

### 4.8. Statistics

The mortality data was entered into an ANOVA to determine the differences among treatments. Post hoc tests were used to measure the differences between means for the mortality counts (α = 0.05). The lethal concentrations for 50 and 90% of the population were computed using a probit analysis (SPSS v.22). The persistence of toxicity was examined by using repeated measures ANOVA, with day as the repeated measures variable, using IBM SPSS for Windows, v.22 (IBM, Armonk, NY, USA). The repellency for *T. castaneum* was tested using a two-way ANOVA, with repellence as the response variable and the concentration and time interval as the main effects.

## 5. Conclusions

BA had significant activity against *T. castaneum* when applied in different ways. BA is safe and FDA-approved for use against lice in humans. In addition, the EFSA (European Food Safety Authority) [67] approved BA in animal feed at a concentration of 125 mg/kg. As a result, it has the potential to be used as an environmentally friendly control against the red flour beetle. More research is needed to explore the BA mode of action and its field stability in the control of this beetle.

## Figures and Tables

**Table 1:** Mortality rate of benzyl alcohol and benzyl benzoate toxicity against *Tribolium castaneum* through impregnated-paper and direct-contact assays after 24 h.

Product Concentration %	Mortality Rate of Benzyl Alcohol Mean ± SD		Mortality Rate of Benzyl Benzoate Mean ± SD	
	Filter Paper Residue	Contact Toxicity	Filter Paper Residue	Contact Toxicity
5	100 ± 0.00 ^a^*	100 ± 0.00 ^a^*	12.7 ± 2.52 ^a^	55.0 ± 5.00 ^a^
2.5	36.7 ± 5.77 ^b^*	53.3 ± 5.77 ^b^*	10.0 ± 1.00 ^b^	33.3 ± 5.77 ^b^
1.25	11.3 ± 1.53 ^c^	40.0 ± 0.00 ^c^*	8.33 ± 1.53 ^b,c^	23.3 ± 5.77 ^c^
0.625	7.67 ± 3.05 ^c,d^	33.3 ± 5.77 ^d^*	7.00 ± 1.00 ^c^	16.6 ± 5.77 ^c^
0.312	7.00 ± 1.00 ^c,d,e^	20.0 ± 0.00 ^e^*	4.33 ± 1.15 ^d^	6.67 ± 5.77 ^d^
0.156	5.67 ± 3.06 ^d,e,f^	8.33 ± 2.88 ^f^	3.66 ± 0.58 ^d^	5.00 ± 5.77 ^d^
0.078	4.66 ± 0.58 ^d,e,f^	6.67 ± 5.77 ^f^	3.33 ± 0.58 ^d^	3.33 ± 5.77 ^d^
0.039	2.67 ± 0.57 ^e,f^	4.67 ± 4.16 ^f^	2.33 ± 0.57 ^d^	3.00 ± 2.64 ^d^
Negative control: acetone	2.00 ± 1.00 ^f^	2.00 ± 1.00 ^f^	2.00 ± 1.00 ^d^	2.00 ± 1.00 ^d^
LC_50_ (95% confidence limits)	2.63 (2.24–3.15)	1.90 (1.40–2.83)	11.7 (7.71–29.6)	4.17 (3.24–6.01)
LC_90_ (95% confidence limits)	4.33 (3.69–5.31)	3.69 (2.78–5.82)	20.1 (12.9–52.7)	7.64 (5.85–11.56)
Regression equation	y = 0.55x − 1.77	y = 0.67x − 1.33	y = 0.15x − 1.76	y = 0.39x − 1.59
Coefficient (R^2^)	0.949	0.768	0.734	0.850

Data are means ± standard deviation (SD); superscript of same letters in cells of the same column indicates non-significance (*p* > 0.05). * Significant difference in comparison to the corresponding group in two different methods (*p* < 0.05).

**Table 2:** Mortality rate of benzyl alcohol and benzyl benzoate toxicity against *Tribolium castaneum* through fumigant assay after 24 h.

Concentrations µL/L Air	Mortality Rate of Fumigation Assay Mean ± SD	
	Benzyl Alcohol	Benzyl Benzoate
300	100 ± 0.00 ^a^*	26.0 ± 5.47 ^a^
150	100 ± 0.00 ^a^*	16.0 ± 5.47 ^a^
75	100 ± 0.00 ^a^*	8.00 ± 1.87 ^c^
35	100 ± 0.00 ^a^*	7.20 ± 2.58 ^c^
20	86.0 ± 5.48 ^b^*	6.60 ± 3.13 ^c^
10	66.0 ± 5.48 ^c^*	6.20 ± 3.56 ^c^
5	40.0 ± 7.07 ^d^*	5.80 ± 3.89 ^c^
2.5	12.0 ± 8.37 ^e^	5.20 ± 4.43 ^c^
Untreated control	4.00 ± 5.47 ^f^	4.00 ± 5.47 ^c^
LC_50_ (95% confidence limits)	6.72 (6.12–7.39)	464 (364–665)
LC_90_ (95% confidence limits)	23.6 (19.9–26.9)	822 (632–1213)
Regression equation	y = 0.12x − 1.08	y = 0.32x − 1.59
R^2^	0.876	0.977

Data are means ± standard deviation (SD); superscript of same letters in the same column indicates non-significance (*p* > 0.05). * Significant difference in comparison to the corresponding group (*p* < 0.05).

**Table 3:** Repeated measures ANOVA parameters for main effects and associated interaction for persistence of toxicity against *Tribolium castaneum*.

Source	Df	Mean Square	*F*	*p*
Day	9	6945.56	352.5	0.0001
Day × Group	18	2262.889	114.846	0.0001
Error	108	19.704		

**Table 4:** Persistence of toxicity as a percentage of LC_90_ for benzyl alcohol and benzyl benzoate against *Tribolium castaneum*.

LC_90_/Time	Benzyl Alcohol, BA (3.69%)	Benzyl Benzoate, BB (7.64%)	Untreated Negative Control
Day 1	100 ± 0.00	100 ± 0.00	0.00 ± 0.00
Day 2	94.0 ± 5.47	62.0 ± 4.47	2.00 ± 4.47
Day 3	88.0 ± 4.47	46.0 ± 5.47	2.00 ± 4.47
Day 4	72.0 ± 4.47	36.0 ± 5.47	4.01 ± 0.43
Day 5	56.0 ± 5.47	22.0 ± 4.47	4.01 ± 0.43
Day 6	44.0 ± 5.47	14.0 ± 5.47	4.10 ± 2.31
Day 7	32.0 ± 4.47	12.0 ± 8.37	4.10 ± 2.42
Day 8	16.0 ± 5.47	6.00 ± 5.47	3.00 ± 1.47
Day 9	6.00 ± 5.47	4.00 ± 5.47	3.00 ± 1.43
Day 10	6.00 ± 5.47	4.00 ± 5.47	3.03 ± 1.01
Mean ± SE	51.4 ± 1.4 ^c^	30.4 ± 1.4 ^b^	3.20 ± 1.4 ^a^

Superscript of different letters in cells of the same row indicates significance (*p* < 0.05).

## Data Availability

All the data related to this work are available in this manuscript.

## References

[1] Hagstrum D., Huang D.W., Klejdysz T.Z., Subramanyam B., Nawrot J. (2017). Infestation records. Atlas of Stored-Product Insects and Mites.

[2] Ajayi F.A., Rahman S.A. (2006). Susceptibility of some staple processed meals to red flour beetle, *Tribolium castaneum* (Herbst)(Coleoptera: Tenebrionidae). Pak. J. Biol. Sci..

[3] El-Mofty M.M., Sakr S.A., Osman S.I., Toulan B.A. (1989). Carcinogenic effect of biscuits made of flour infested with *Tribolium castaneum* in Buforegularis. Oncology.

[4] Shafique M., Ahmad M., Chaudry M.A. (2006). Feeding preference and development of *Tribolium castaneum* (Herbst.) in wheat products. Pak. J. Zool..

[5] Fedina T.Y., Lewis S.M. (2008). An integrative view of sexual selection in Tribolium flour beetles. Biol. Rev..

[6] Bergerson O., Wool D. (1988). The process of adaptation of flour beetles to new environments. Genetica.

[7] Arnaud L., Brostaux Y., Lallemand S., Haubruge E. (2005). Reproductive strategies of Tribolium flour beetles. J. Insect Sci..

[8] Bell C.H. (2000). Fumigation in the 21st century. Crop Protect..

[9] Carter C.A., Chalfant J.A., Goodhue R.E., Han F.M., DeSantis M. (2005). The methyl bromide ban: Economic impacts on the California strawberry industry. Rev. Agric. Econ..

[10] Anbar A.D., Yung Y.L., Chavez F.P. (1996). Methyl bromide: Ocean sources, ocean sinks, and climate sensitivity. Glob. Biogeochem. Cycles.

[11] Benhalima H., Chaudhry M.Q., Mills K.A., Price N.R. (2004). Phosphine resistance in stored-product insects collected from various grain storage facilities in Morocco. J. Stored Prod. Res..

[12] Okwute S.K. (2012). Plants as potential sources of pesticidal agents: A review. Pestic. Adv. Chem. Bot. Pestic..

[13] Chaudhary S., Kanwar R.K., Sehgal A., Cahill D.M., Barrow C.J., Sehgal R., Kanwar J.R. (2017). Progress on *Azadirachta indica* based biopesticides in replacing synthetic toxic pesticides. Front. Plant Sci..

[14] Pugh S., McKenna R., Halloum I., Nielsen D.R. (2015). Engineering *Escherichia coli* for renewable benzyl alcohol production. Metab. Eng. Commun..

[15] Ash M., Ash I. (2009). Handbook of Preservatives. Synapse Information Resources.

[16] Stellman J.M. (1998). Encyclopaedia of Occupational Health and Safety.

[17] Stoye D., Werner F. (2008). Paints, Coatings and Solvents.

[18] Nair B. (2001). Final report on the safety assessment of Benzyl Alcohol, Benzoic Acid, and Sodium Benzoate. Int. J. Toxicol..

[19] Fenaroli G., Burdock G.A. (1995). Handbook of Flavor Ingredients.

[20] Marriott J.F. (2010). Pharmaceutical Compounding and Dispensing.

[21] Felton L. (2013). Remington Essentials of Pharmaceutics.

[22] Meinking T.L., Villarm M.E., Vicaria M., Eyerdam D.H., Paquet D., Mertz-Rivera K., Rivera H.F., Hiriart J., Reyna S. (2010). The clinical trials supporting benzyl alcohol lotion 5% (Ulesfia): A safe and effective topical treatment for head lice (*Pediculosis humanus capitis*). Pediatr. Dermatol..

[23] Budavari S., O’Neil M., Smith A., Heckelman P. (1989). The Merck Index: An Encyclopedia of Chemicals, Drug, and Biologicals.

[24] Aboelhadid S.M., Ibrahium S.M., Abdel-Baki A.S., Hassan K.M., Arafa W.M., Aboud H.M., Mohy S., Al-Quraishy S., Hassan A.O., Abdelgelil N.H. (2023). An investigation of the acaricidal activity of benzyl alcohol on *Rhipicephalus annulatus and Rhipicephalus sanguineus* and its synergistic or antagonistic interaction with commonly used acaricides. Med. Vet. Entomol..

[25] Sangaré A.K., Doumbo O.K., Raoult D. (2016). Management and Treatment of Human Lice. Biomed. Res. Int..

[26] Johnson W., Bergfeld W.F., Belsito D.V., Hill R.A., Klaassen C.D., Liebler D.C., Marks J.G., Shank R.C., Slaga T.J., Snyder P.W. (2017). Safety Assessment of Benzyl Alcohol, Benzoic Acid and its Salts, and Benzyl Benzoate. Int. J. Toxicol..

[27] Pearson M.A., Miller G.W. (2014). Encyclopedia of Toxicology.

[28] Stuart M.C., Kouimtzi M., Hill S.R., World Health Organization (2009). WHO Model Formulary 2008.

[29] Harju A.T., Pennanen S.M., Liesivuori J. (2004). The efficacy of benzyl benzoate sprays in killing the storage mite *Tyrophagus putrescentiae* (Acari: Acaridae). Ann. Agric. Environ. Med..

[30] Chang J.H., Becker A., Ferguson A., Manfreda J., Simons E., Chan H., Noertjojo K., Chan-Yeung M. (1996). Effect of application of benzyl benzoate on house dust mite allergen levels. Ann. Allergy. Asthma Immunol..

[31] Raynaud S., Fourneau C., Laurens A., Hocquemiller R., Loiseau P., Bories C. (2000). Squamocin and benzyl benzoate, acaricidal components of Uvaria pauci-ovulata bark extracts. Planta Med..

[32] Kalpaklioğlu A.F., Ferizli A.G., Misirligil Z., Demirel Y.S., Gürbüz L. (1996). The effectiveness of benzyl benzoate and different chemicals as acaricides. Allergy.

[33] Forton F.M.N., De Maertelaer V. (2020). Treatment of rosacea and demodicosis with benzyl benzoate: Effects of different doses on Demodex density and clinical symptoms. J. Eur. Acad. Dermatol. Venereol..

[34] Forton F.M.N., De Maertelaer V. (2022). Effectiveness of benzyl benzoate treatment on clinical symptoms and Demodex density over time in patients with rosacea and demodicosis: A real life retrospective follow-up study comparing low- and high-dose regimens. J. Dermatol. Treat..

[35] Forton F., Seys B., Marchal J.L., Song M. (1998). Demodex folliculorum and topical treatments: Acaricide action evaluated by standardized skin-surface biopsy. Br. J. Dermatol..

[36] Page S.W. (2008). Small Animal Clinical Pharmacology.

[37] Smith L.W., Pratt I.N.I.I., Umina A.P. (1971). Baking and taste properties of bread made from hard wheat flour infested with species Tribolium, Tenebrio, Trogoderma and Oryzaphilus. J. Stored Prod. Res..

[38] Boyer S., Zhang H., Lemperiere G. (2012). A review of control methods and resistance mechanisms in stored-product insects. Bull. Entomol. Resour..

[39] Guedes R.N.C. (1990). Manejo integrado para a protecao de graos armazenados contra insetos. Review BrasilArmazen. Veg. Sobre Triboliumcastaneum Herbst. Agriscientia.

[40] Brower J.H., Smith L., Vail P.V., Flinn P.W., Subramanyam B.H., Hagstrum D.W. (1996). Biological Control. Integrated Management of Insect in Stored Products Pest.

[41] Gottschalck T.E., Bailey J.E. (2010). International Cosmetic Ingredient Dictionary and Handbook.

[42] Acar A., Turkmen Z., Cavusoglu K., Yalcin E. (2020). Investigation of benzyl benzoate toxicity with anatomical, physiological, cytogenetic and biochemical parameters in in vivo. Caryologia.

[43] Brooks P.A., Grace R.F. (2002). Ivermectin is better than benzyl benzoate for childhood scabies in developing countries. J. Paediatr. Child Health.

[44] McDonald L.G., Tovey E. (1993). The effectiveness of benzyl benzoate and some essential plant oils as laundry additives for killing house dust mites. J. Allergy Clin. Immunol..

[45] Jantan I.B., Yalvema M.F., Ahmad N.W., Jamal J.A. (2005). Insecticidal activities of the leaf oils of eight *Cinnamomum.* species against *Aedes aegypti.* and *Aedes Albopictus*. Pharm. Biol..

[46] Diastuti H., Chasani M., Suwandri S. (2020). Antibacterial Activity of Benzyl Benzoate and Crotepoxide from Kaempferia rotunda L. Rhizome. Indones. J. Chem..

[47] Suhaili Z., Ho T. (2008). Residual Activity of Benzyl Benzoate Against *Dermatophagoides Pteronyssinus* (Acari: Pyroglyphidae). Southeast Asian J. Trop. Med. Public Health.

[48] Monteiro I.N., Monteiro O.D.S., Costa-Junior L.M., da Silva Lima A., Andrade E.H.A., Maia J.G.S., Mouchrek Filho V.E. (2017). Chemical composition and acaricide activity of an essential oil from a rare chemotype of Cinnamomum verum Presl on *Rhipicephalus microplus* (Acari: Ixodidae). Vet. Parasitol..

[49] Deb M., Kumar D. (2020). Bioactivity and efficacy of essential oils extracted from *Artemisia annua* against *Tribolium castaneum* (Herbst. 1797) (Coleoptera: Tenebrionidae): An eco-friendly approach. Ecotoxicol. Environ. Saf..

[50] Rajashekar Y., Raghavendra A., Bakthavatsalam N. (2014). Acetylcholinesterase inhibition by biofumigant (Coumaran) from leaves of *Lantana camara* in stored grain and household insect pests. BioMed Res. Int..

[51] Cohen E. (1986). Glutathione S transferase activity and its induction in several strains of *Tribolium castaneum*. Entomol. Exp. Appl..

[52] Hasspieler B.M., Arnason J.T., Downe A.E.R. (1990). Modes of action of the plant-derived phototoxin α-terthienyl in mosquito larvae. Pestic. Biochem. Physiol..

[53] Yano T., Miyahara Y., Morii N., Okano T., Kubota H. (2015). Pentanol and Benzyl Alcohol Attack Bacterial Surface Structures Differently. Appl. Environ. Microbiol..

[54] Aboelhadid S.M., Youssef I.M. (2021). Control of red flour beetle (*Tribolium castaneum*) in feeds and commercial poultry diets via using a blend of clove and lemongrass extracts. Environ. Sci. Pollut. Res..

[55] Kljajic P., Peric I. (2006). Susceptibility to contact insecticides of granary weevil *Sitophilus granarius* (L.) (Coleoptera: Curculionidae) originating from different locations in the former Yugoslavia. J. Stored Prod. Res..

[56] Busvine J.R. (1980). Recommended Methods for Measurement of Pest Resistance to Pesticides.

[57] Hashem A.S., Awadalla S.S., Zayed G.M., Maggi F., Benelli G. (2018). *Pimpinella anisum* essential oil nanoemulsions against *Tribolium castaneum* insecticidal activity and mode of action. Environ. Sci. Pollut. Res..

[58] Abouelatta A.M., Keratum A.Y., Ahmed S.I., El-Zun H.M. (2020). Repellent, contact and fumigant activities of geranium (*Pelargonium graveolens* L.’Hér) essential oils against *Tribolium castaneum* (Herbst) and *Rhyzopertha dominica* (F.). Int. J. Trop. Insect Sci..

[59] Lak F., Zandi-Sohani N., Ghodoum Parizipour M.H., Ebadollahi A. (2022). Synergic effects of some plant-derived essential oils and Iranian isolates of entomopathogenic fungus *Metarhizium anisopliae* Sorokin to control *Acanthoscelides obtectus* (Say) (Coleoptera: Chrysomelidae). Front. Plant Sci..

[60] Ilboudo Z., Dabiré L.C.B., Nébié R.C.H., Dicko I.O., Dugravot S., Cortesero A.M., Sanon A. (2010). Biological activity and persistence of four essential oils towards the main pest of stored cowpeas, *Callosobruchus maculatus* (F.) (Coleoptera: Bruchidae). J. Stored Prod. Res..

[61] Cosimi S., Rossi E., Cioni P.L., Canale A. (2009). Bioactivity and qualitative analysis of some essential oils from Mediterranean plants against stored-product pests: Evaluation of repellency against *Sitophilus zeamais Motschulsky*, *Cryptolestes ferrugineus* (Stephens) and *Tenebrio molitor* (L.). J. Stored Prod. Res..

[62] Cardoso A.D.S., Santos E.G.G., Lima A.D.S., Temeyer K.B., Pérez de León A.A., Costa L.M., Junior Soares A.M.D.S. (2020). Terpenes on *Rhipicephalus* (*Boophilus*) *microplus*: Acaricidal activity and acetylcholinesterase inhibition. Vet. Parasitol..

[63] Ellman G.L., Courtney K.D., Andres J.R.V., Featherstone R.M. (1961). A new and rapid colorimetric determination of acetylcholinesterase activity. Biochem. Pharmacol..

[64] Anderson J.A., Coats J.R. (2011). Acetylcholinesterase inhibition by nootkatone and carvacrol in arthropods. Pestic. Biochem. Physiol..

[65] Tavares C.P., Sabadin G.A., Sousa I.C., Gomes M.N., Soares A.M., Monteiro C.M., Costa-Junior L.M. (2022). Effects of carvacrol and thymol on the antioxidant and detoxifying enzymes of *Rhipicephalus microplus* (Acari: Ixodidae). Ticks Tick Borne Dis..

[66] Bar-Or D., Rael L.T., Lau E.P., Rao N.K., Thomas G.W., Winkler J.V., Yukl R.L., Kingston R.G., Curtis C.G. (2001). An analog of the human albumin N-terminus (Asp-Ala-His-Lys) prevents formation of copper-induced reactive oxygen species. Biochem. Biophys. Res. Commun..

[67] EFSA (European Food Safety Authority) (2012). Scientific Opinion on the safety and efficacy of benzyl alcohols, aldehydes, acids, esters and acetals (chemical group 23) when used as flavourings for all animal species. EFSA J..

